# Long-Term Comparative Efficacy and Safety of Different Patent Foramen Ovale Closure Devices: Real-World Evidence from a 15-Year Cohort

**DOI:** 10.3390/jcm15093429

**Published:** 2026-04-30

**Authors:** Zeynep Yapan Emren, Sadık Volkan Emren, Uğur Karagöz, Aykan Çelik, Emre Özdemir, Fahrettin Tuğrul Çitekçi, Semih Aktürk, Bahadır Akar

**Affiliations:** 1 Department of Cardiology, Bakırçay University Çiğli Training and Research Hospital, Izmir 35620, Türkiye; 2 Department of Cardiology, Katip Çelebi University Atatürk Training and Research Hospital, Izmir 35360, Türkiye; vemren@hotmail.com (S.V.E.); ugur.karagoz@ikc.edu.tr (U.K.);

**Keywords:** Patent Foramen Ovale, stroke, atrial fibrillation

## Abstract

**Objectives:** Percutaneous Patent Foramen Ovale (PFO) closure has become a standard treatment for secondary prevention following cryptogenic stroke. This study aims to compare the long-term clinical outcomes and stroke recurrence rates between the Amplatzer PFO Occluder and other commercially available devices, including Occlutech, Cardiofix, and Biostar. **Methods:** This retrospective study included 130 consecutive patients who underwent percutaneous PFO closure due to PFO-related stroke. The participants were divided into two groups: the Amplatzer group (n = 90, 69%) and the Other Devices group. Primary endpoints were Major Adverse Cardiovascular Events (MACE) defined as stroke recurrence, new-onset atrial fibrillation (AF), and mortality during the follow-up period. **Results:** The average follow-up period was 2200 days (median 1739 days, interquartile range: 1062–2616 days). No statistically significant differences were observed between the groups regarding baseline characteristics such as age, hypertension, or diabetes. The Amplatzer and other device groups showed similar rates for MACE, atrial fibrillation, and stroke recurrence. Kaplan–Meier analysis demonstrated no significant difference in event-free survival between the groups. This study demonstrates that the Amplatzer PFO Occluder and other commercial devices provide comparable long-term safety and efficacy profiles in patients undergoing PFO closure for cryptogenic stroke. Regardless of the device type used, long-term stroke recurrence rates remain low in both groups.

## 1. Introduction

Cryptogenic stroke (CS) remains a significant clinical challenge, accounting for approximately 30% to 40% of all ischemic strokes [1]. A strong association exists between CS and the presence of a Patent Foramen Ovale (PFO), a condition found in 20% to 25% of the general adult population but in 40% to 60% of cryptogenic stroke patients [2,3].

Recent randomized controlled trials (RCTs) have provided robust evidence that percutaneous PFO closure offers superior secondary stroke prevention compared to medical therapy alone, particularly in selected patients [4,5,6,7]. However, while this meta-analysis focuses on the broad comparison between closure and medication, there remains a critical need for long-term, head-to-head comparisons between specific commercial occluder devices in real-world settings.

The evolution of transcatheter PFO closure has involved the use of various devices, underscoring the necessity of comparative effectiveness studies. Given that the benefit of PFO closure is expected to accrue over time, often extending well beyond 5 years, long-term comparative data on device performance are critical for clinical decision-making [8]. Shorter-term or similar-duration cohorts have evaluated specific device pairs, such as Amplatzer vs. Occlutech [9]. More recently, long-term echocardiographic features and structural integrity following percutaneous closure further highlight the necessity for ongoing monitoring of device performance. However, there is still a paucity of real-world data for less frequently reported devices such as Cardiofix and Biostar, moving beyond the standard Amplatzer vs. Occlutech comparison in a consistent, long-term 15-year cohort. This study justifies its novelty by providing extended follow-up data to validate whether the findings from larger registries or different geographic cohorts remain consistent in our specific patient population.

This study aims to address this critical knowledge gap by comprehensively comparing the long-term stroke recurrence rates among patients treated with the Amplatzer PFO Occluder against those treated with other commercially available devices used for percutaneous PFO closure.

## 2. Materials and Methods

This retrospective study included 130 consecutive patients who underwent percutaneous PFO closure due to cryptogenic or PFO-related stroke after detailed neurological evaluation. The data of the patients were obtained retrospectively. The study is described as a ‘15-year cohort’ to reflect the total longitudinal duration of the study period, spanning from the inclusion of the first patient in 2008 to the final follow-up evaluations in 2023. In contrast, the reported mean and median follow-up durations (2200 and 1739 days, respectively) represent the actual per-patient observation time. This distinction arises because patients were enrolled consecutively over a 12-year period (2008–2020), resulting in varying individual follow-up lengths depending on their date of procedure.

This study was conducted in accordance with the principles of the Declaration of Helsinki. Ethical approval was obtained from the institutional review board (Katip Çelebi University Ethics Committee, Approval code: 0474, Approval date: 17 July 2025), and informed consent was obtained from all participants prior to inclusion in the study.

Patients with a history of stroke or transient ischemic attack, age between 18–65 years, detection of PFO with moderate or significant shunting defined as the presence of more than 20 microbubbles in the left atrium within three cardiac cycles after complete opacification of the right atrium during a standardized valsalva maneuver, as evaluated by transesophageal echocardiography, and/or atrial septal aneurysm defined as a protrusion of the septal tissue into either the right or left atrium of >10 mm from the vertical plane of the septum, or a total excursion (sum of both sides) of >15 mm, large PFO (2 mm and more separation between septum primum and secundum), and septal hypermobility under transesophageal echocardiography were included in the study [3].

Patients with significant atherosclerosis, atrial fibrillation, arterial dissection, lacunar infarct, rheumatological disease, malignancy, and intracranial aneurysm were excluded from the study.

The 130 patients were divided into two main intervention groups; the first group was the Amplatzer PFO Occluder Group: patients treated with the Amplatzer PFO Occluder (St. Jude Medical/Abbott). The second group was the other devices group: patients treated with alternative commercially available PFO closure devices (e.g., FIGULLA, BIOSTAR, CARDIOFIX).

All procedures were performed percutaneously using standard interventional techniques, usually guided by fluoroscopy and transesophageal echocardiographic guidance (TEE). Device deployment success required achieving stable placement across the atrial septum. Effective closure was defined as complete occlusion or clinically insignificant residual shunting confirmed during post-procedural imaging.

The Risk of Paradoxical Embolism (ROPE) Score was calculated during the index event. All patients’ digital medical databases were searched in terms of mortality, stroke events, and AF. The primary endpoint was defined as stroke, atrial fibrillation, and mortality during follow-up. The composite endpoint of MACE—comprising stroke recurrence, new-onset atrial fibrillation (AF), and all-cause mortality—was utilized to provide a comprehensive assessment of the long-term clinical safety and efficacy of PFO closure. Combining these distinct events is clinically justified as they represent the most significant cardiovascular burdens following PFO-related stroke. While stroke recurrence directly measures procedural success in secondary prevention, new-onset AF is a critical device-related complication that significantly increases the future risk of thromboembolic events. Mortality was included to ensure a complete representation of the patients’ ultimate clinical status. Using a composite endpoint increases the statistical power of the study to detect overall differences in clinical outcomes between device groups in a cohort where individual event rates, such as recurrent stroke, are expected to be low.

Statistical analyses were performed using jamovi (Version 2.3 or higher). Continuous variables were expressed as mean ± standard deviation (SD) for normally distributed data, and as median with interquartile range (IQR) for non-normally distributed data. Categorical variables were presented as frequencies and percentages (N/%).

The normality of the data was assessed using the Shapiro–Wilk test. For the comparison of continuous variables between the Amplatzer and other device groups, the Independent Samples *t*-test or Mann–Whitney U test was utilized where appropriate. Categorical data were compared using the Chi-square test or Fisher’s exact test.

Follow-up duration was calculated by converting the follow-up days into months (Days/30). Survival curves and event-free survival rates were estimated using the Kaplan–Meier method, and the differences between device groups were evaluated using the Log-rank (Mantel-Cox) test.

To identify potential predictors of clinical events, Cox Regression analyses were conducted. Results were reported as Hazard Ratios (HR) with 95% Confidence Intervals (CI). A *p*-value of <0.05 was considered statistically significant for all analyses.

**Flow chart of the study:**


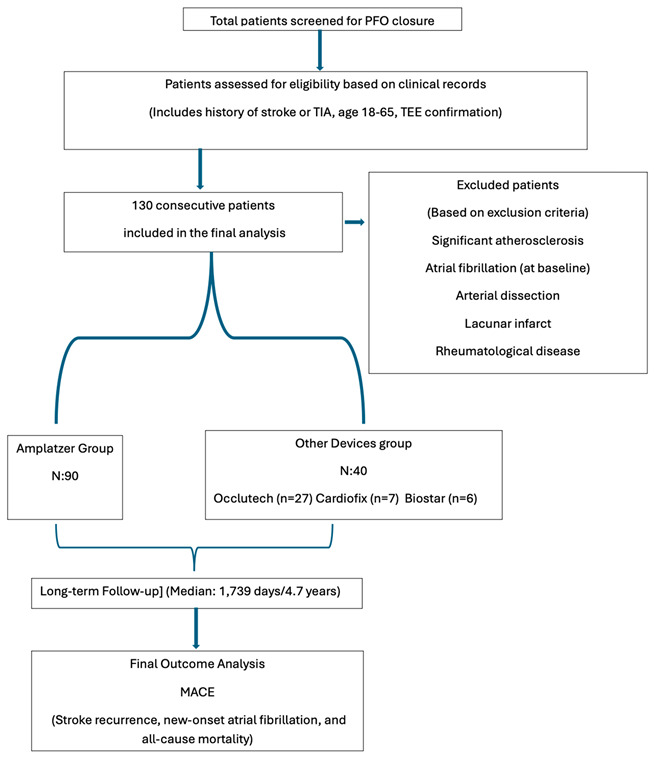


## 3. Results

**Figure 1:**
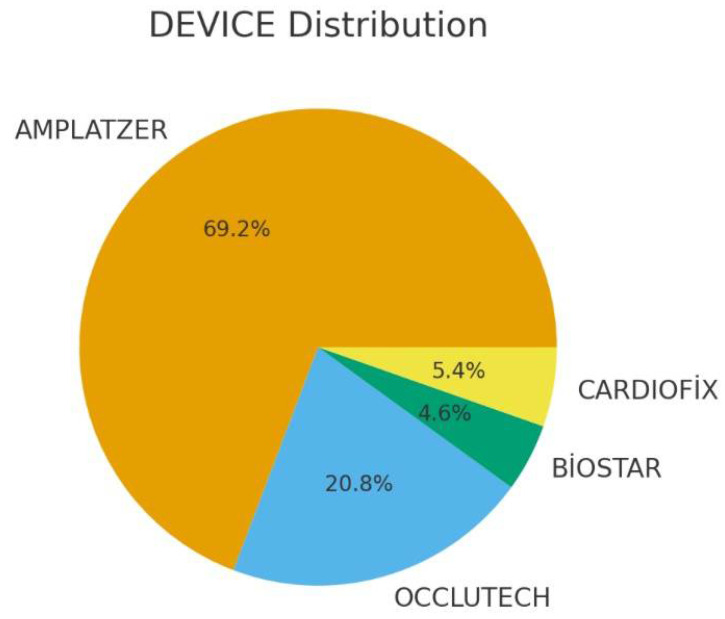
Distribution of Patent Foramen Ovale (PFO) closure devices. This pie chart illustrates the proportional distribution of the different occluder devices used in the study cohort (N = 130). The Amplatzer PFO Occluder was the most utilized device (69.2%, N = 90), followed by Occlutech (20.8%, n = 27), Cardiofix (5.4%, n = 7), and Biostar (4.6%, n = 6). Data are presented as percentages and absolute patient counts.

The average follow-up period was a mean of 2200 days (median 1739 days, interquartile range: 1062–2616 days). A total of 130 patients who underwent PFO closure were included in the study. The Amplatzer PFO Occluder was the most frequently used device (69.2%), followed by Occlutech (20.8%), Cardiofix (5.4%), and Biostar (4.6%) (Figure 1). The mean age was 43 ± 11.6 years in the Amplatzer group and 45 ± 11.1 years in the other devices group (*p* = 0.272). There were no statistically significant differences between the two groups regarding comorbidities, including hypertension (*p* = 0.213), diabetes (*p* = 0.714), and smoking (*p* = 0.411) (Table 1).

**Figure 2:**
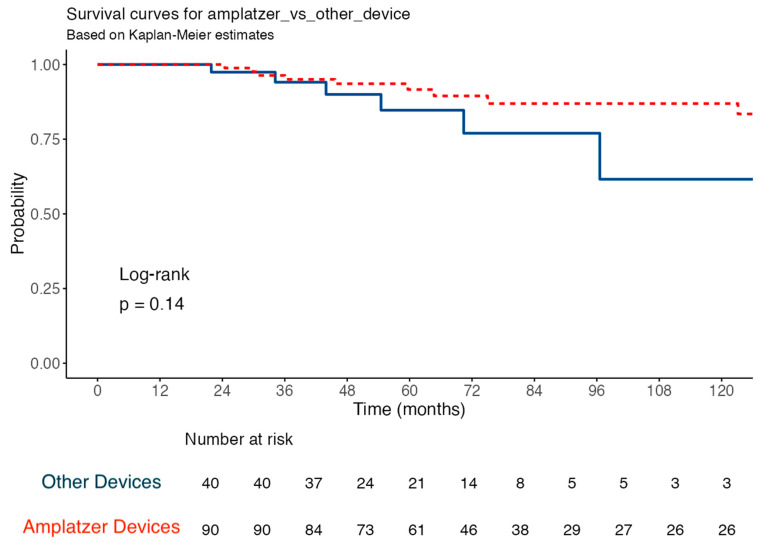
Kaplan–Meier estimates of event-free survival by device group.

**Figure 3:**
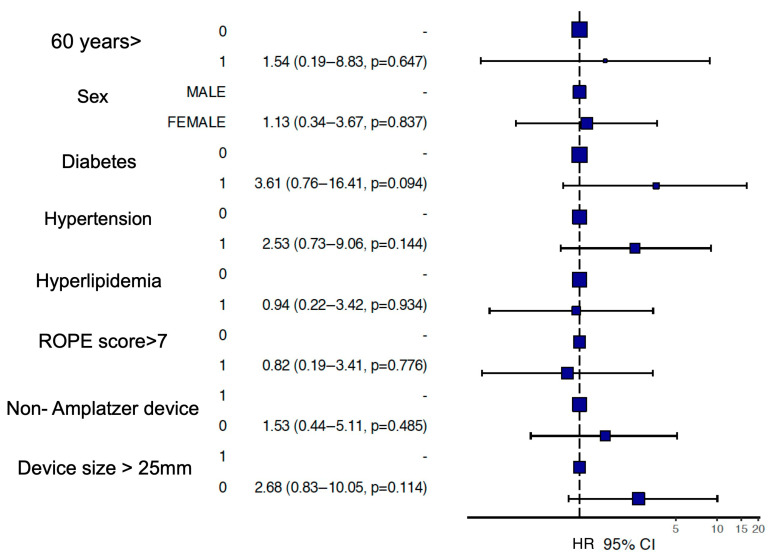
Forest plot of subgroup analysis for clinical outcomes.

The median occluder size was similar between groups (30 mm (19–30 mm) for Amplatzer vs. 25 mm (25–30 mm) for others, *p* = 0.905). Amplatzer and other device groups had similar MACE (9 (7%) vs. 6 (5%) *p* = 0.410), atrial fibrillation (4 (3%) vs. 1 (1%) *p* = 0.595), and stroke (2 (1.5%) vs. 3 (2.3%) *p* = 0.149) rates during the follow-up period. Kaplan–Meier survival analysis demonstrated no significant difference in event-free survival between the Amplatzer and non-Amplatzer groups over a 5-year follow-up period (Log-rank *p* = 0.14) (Figure 2). In the subgroup analysis, none of the evaluated parameters were found to be independent predictors of outcomes (Figure 3).

Comparison of long-term composite primary endpoint between patients treated with the Amplatzer PFO Occluder (n = 90) and alternative ‘Other’ devices (n = 40). The composite primary endpoint (MACE) included stroke recurrence, new-onset atrial fibrillation, and all-cause mortality during the follow-up period. Kaplan–Meier analysis demonstrates no statistically significant difference in event-free survival between the two groups over a 120-month follow-up period (Log-rank *p* = 0.14). The ‘Number at Risk’ table at the bottom shows the number of patients remaining in the follow-up at each 12-month interval.

This forest plot displays the results of the Cox regression analysis conducted to identify potential predictors of clinical events during the follow-up period. Risk factors are stratified by the presence (bottom) versus the absence (top) of each factor to calculate the respective confidence intervals. The analysis evaluates various parameters, including age (>60 years), sex, comorbidities (diabetes, hypertension, hyperlipidemia), ROPE score (>7), device type (Non-Amplatzer), and device size (>25 mm). Hazard ratios (HR) with 95% Confidence Intervals (CI) and corresponding *p*-values are provided for each subgroup. No parameters were found to be independent predictors of adverse outcomes.

## 4. Discussion

This study aimed to compare the long-term clinical outcomes between the Amplatzer PFO Occluder and other commercially available devices (Occlutech, Cardiofix, Biostar) in patients who underwent percutaneous PFO closure due to cryptogenic stroke or PFO-related stroke. Our findings demonstrate that over a 5-year follow-up period, there were no significant differences between the Amplatzer device and other devices regarding stroke recurrence, atrial fibrillation, and major adverse cardiovascular events.

These findings are consistent with comparative studies in the current literature. A recent study from Switzerland based on the SOLUTION registry compared the Amplatzer PFO Occluder with the Cardia PFO device and found no significant difference in the primary endpoint (stroke, TIA, or peripheral embolism) in an analysis of 934 patients (4% in Amplatzer vs. 2% in Cardia, *p* = 0.20) [10]. Both devices were equally effective in preventing recurrent events. Another study found that the bubble transition and the stroke rate 52 months after intervention were similar between the Amplatzer and Occlutech groups [8]. A prospective analysis comparing Cardi-O-fix and Amplatzer devices reported no significant differences in complete closure rates (90.6% vs. 86.4%) or procedural complications during mid-term follow-up [11].

Similarly, in a 23-year monocentric analysis, Kayvanpour and colleagues compared Occlutech, Amplatzer, Gore septal occluder, and Cardia PFO-Star devices, and found no significant differences in procedural success rates (98.9%) or complication rates between devices [12]. This study also demonstrates that different devices have similar safety and efficacy profiles.

The long-term efficacy of the Amplatzer PFO Occluder observed in our study aligns with the low event rates reported in the literature. In a recent post-marketing surveillance study from Japan involving 500 patients with the Amplatzer device, the ischemic stroke rate was 0.6% and the atrial fibrillation rate was 2.4% at 1-year follow-up [13]. These rates are consistent with the long-term event rates in our study and support the low complication profile of the Amplatzer device. Long-term data from the Amplatzer PFO Occluder registry with up to 10 years of follow-up demonstrated durable efficacy, with annual stroke rates of 1.5% [14].

Atrial fibrillation (AF) following PFO closure is a well-recognized complication in the literature [15,16]. The incidence of new-onset AF is often associated with several key predictors, including advanced age, male gender, and the presence of cardiovascular comorbidities such as hypertension and obesity. Anatomical factors, particularly left atrial enlargement and the presence of an atrial septal aneurysm (ASA), also play a significant role in increasing arrhythmogenic potential. Furthermore, procedural elements such as device size and the specific device design can influence the mechanical tension on the atrial septum, potentially triggering early-onset AF [17,18,19]. The lack of difference in AF incidence between the two groups in our study suggests that both device types have similar arrhythmogenic potential. None of the above factors were associated with AF in our study.

We explicitly acknowledge that the retrospective and non-randomized design of this study is a primary limitation that introduces inherent selection bias. The allocation of specific PFO closure devices was not randomized and was instead influenced by temporal availability, operator preference, and evolving institutional protocols over the 15-year study period. Second, our sample size is relatively small, which may result in being underpowered to detect subtle between-group differences in rare clinical events, such as recurrent stroke and new-onset atrial fibrillation. Given the low overall incidence of these events in both the Amplatzer and alternative device groups, the lack of statistical significance should be interpreted with caution, as it does not definitively exclude the possibility of a smaller clinical difference that might only be detectable in a much larger, multicenter cohort. Third, atrial fibrillation follow-up was not performed with continuous rhythm monitoring, and subclinical AF episodes may have been missed. Fourth, the “other devices” group includes multiple device types with different characteristics, and the inability to analyze each device separately represents an important limitation. Fifth, the lack of systematic evaluation of procedural success parameters, such as residual shunt presence and degree during long-term follow-up, complicates the complete interpretation of clinical outcomes. Finally, medication adherence and differences in antithrombotic treatment regimens were not controlled in our study, and these factors may have influenced clinical outcomes.

The novelty of this study lies in its 15-year cohort span (2008–2023) and an exceptionally long follow-up period, with a mean of 2200 days and a median of approximately 4.7 years. While existing literature often focuses on short-to-mid-term outcomes or limited head-to-head comparisons between the two most common devices, our study provides robust ‘real-world’ evidence by evaluating a diverse mix of occluders, including Cardiofix and Biostar. These data are critical because the clinical benefits of PFO closure are known to accrue over time, and our long-term findings reinforce the sustained safety and efficacy of the procedure across various device platforms in a specific regional population.

## 5. Conclusions

This study demonstrates that in patients undergoing PFO closure for cryptogenic stroke, the Amplatzer PFO Occluder and other commercial devices yield similar outcomes regarding stroke recurrence, atrial fibrillation, and MACE over 5 years of follow-up.

## Figures and Tables

**Table 1:** Demographic table.

	Amplatzer PFO Occluder N: 90	None-Amplatzer PFO Occluder Devices N: 40	*p* Value
Age, years mean ± sd	43 ± 11.6	45 ± 11.1	0.272
Female N (%)	35 (27%)	22 (17%)	0.088
Hypertension N (%)	24 (18%)	15 (11%)	0.213
Diabetes N (%)	11 (8%)	4 (3%)	0.714
Smoking N (%)	43 (33%)	16 (12%)	0.411
Hemoglobin (g/dL) Median (IQR)	14 (12–15)	14 (11–14)	0.046 *
Platelet (10^3^/µL) Median (IQR)	258 (212–292)	267 (230–311)	0.279 *
Creatinine mg/dL Median (IQR)	0.82 (0.73–0.93)	0.78 (0.70–0.90)	0.050 *
Total cholesterol mg/dL	185 (155–211)	171 (139–212)	0.147 *
ROPE score Median (IQR)	7 (5–8)	6 (6–7)	0.898 *
Cortical infarct N (%)	33 (25%)	20 (15%)	0.153
Coagulopathy N (%)	6 (5%)	3 (2%)	0.790
Occluder Size Median (IQR)	30 (19–30)	25 (25–30)	0.905 *
MACE Median (IQR)	9 (7%)	6 (5%)	0.410
Atrial fibrillation N (%)	4 (3%)	1 (1%)	0.595
Stroke N (%)	2 (1.5%)	3 (2.3%)	0.149
Mortality N (%)	4 (3%)	3 (2%)	0.476

IQR: Interquartile range, Mann-Whitney U test, PFO: Patent Foramen Ovale, ROPE: Risk of Paradoxical Embolism, MACE: Major Adverse Cardiac Events, N: Number, sd: standard deviation, *: Whitney U test.

## Data Availability

The data presented in this study are available on request from the corresponding author due to (specify the reason for the restriction).
